# *Plasmodium*—a brief introduction to the parasites causing human malaria and their basic biology

**DOI:** 10.1186/s40101-020-00251-9

**Published:** 2021-01-07

**Authors:** Shigeharu Sato

**Affiliations:** 1 Borneo Medical and Health Research Centre, Faculty of Medicine and Health Sciences, Universiti Malaysia Sabah, Jalan UMS, 88400 Kota Kinabalu, Sabah Malaysia; 2 Department of Pathobiology and Medical Diagnostics, Faculty of Medicine and Health Sciences, Universiti Malaysia Sabah, Jalan UMS, 88400 Kota Kinabalu, Sabah Malaysia

**Keywords:** Antimalarial, Asymptomatic carrier, Drug resistance, Host specificity, Host switch, Malaria, Mosquito, *Plasmodium*, Recurrence, Zoonosis

## Abstract

Malaria is one of the most devastating infectious diseases of humans. It is problematic clinically and economically as it prevails in poorer countries and regions, strongly hindering socioeconomic development. The causative agents of malaria are unicellular protozoan parasites belonging to the genus *Plasmodium.* These parasites infect not only humans but also other vertebrates, from reptiles and birds to mammals. To date, over 200 species of *Plasmodium* have been formally described, and each species infects a certain range of hosts. *Plasmodium* species that naturally infect humans and cause malaria in large areas of the world are limited to five—*P. falciparum*, *P. vivax*, *P. malariae*, *P. ovale* and *P. knowlesi*. The first four are specific for humans, while *P. knowlesi* is naturally maintained in macaque monkeys and causes zoonotic malaria widely in South East Asia. Transmission of *Plasmodium* species between vertebrate hosts depends on an insect vector, which is usually the mosquito. The vector is not just a carrier but the definitive host, where sexual reproduction of *Plasmodium* species occurs, and the parasite’s development in the insect is essential for transmission to the next vertebrate host. The range of insect species that can support the critical development of *Plasmodium* depends on the individual parasite species, but all five *Plasmodium* species causing malaria in humans are transmitted exclusively by anopheline mosquitoes. *Plasmodium* species have remarkable genetic flexibility which lets them adapt to alterations in the environment, giving them the potential to quickly develop resistance to therapeutics such as antimalarials and to change host specificity. In this article, selected topics involving the *Plasmodium* species that cause malaria in humans are reviewed.

## Background: battle of humans against malaria—past and present

Malaria has been recognised as a serious health problem since the earliest historical times. This disease is caused by protozoan parasites belonging to the genus *Plasmodium*. The strong negative pressure of the disease has likely forced the evolution of human populations in malaria endemic regions and the selection of some unique genetic variants. For example, thalassemia and sickle-cell disease, each of which is a genetic disorder affecting red blood cells, are commonly found in malaria endemic areas [1], and people with these two disorders show resistance to malaria. Another well-known example is the Duffy-negative blood type that the majority of people living in Central and West Africa have [2]. This confers specific resistance to infection by one particular *Plasmodium* species, *P. vivax* [3, 4]. The spread of this trait in the population is estimated to have begun around 42,000 years ago [5], and today *P. vivax* malaria is rare in these areas whereas *P. falciparum* malaria is prevalent [6].

Even in the modern world with effective antimalarials and insecticide-treated bed nets (ITNs), people in many countries remain at risk, and the number of malaria cases, particularly those resulting from *P. falciparum* that causes the most serious infection, remains high in economically poor countries, especially in Africa [7]. Rolling out adequate and continuous programmes to effectively control malaria has been difficult mainly due to lack of finance. In 2000, the health programme to “combat malaria” was selected as one of the critical global targets of the Millennium Development Goals set by the United Nations. This global effort was to achieve the targets that were set for measurable health indicators, such as disease prevalence, death rates and protection of children under 5-years of age with ITNs and appropriate antimalarial drugs. In 2005, the sum of global investments for malaria control was an estimated US$960 million, mostly from National Malaria Control Programmes (NMCPs) [7]. Whilst contributions from NMCPs remained at the same level, investments from other sources started to increase steadily since 2006. As a result, the sum of global investments surpassed US$2000 million in 2009 and has remained almost at this level thereafter [7]. With this extra financing, malaria control programs achieved a remarkable level of progress globally. For example, in 2000, the annual deaths caused by malaria in the entire world and in Africa were estimated to be 839,000 (between 653,000 and 1.1 million) and 694,000 (569,000–901,000), respectively. By 2015, these numbers had been reduced to 483,000 (236,000–635,000) and 292,000 (212,000–384,000) [7]. The number of countries estimated to have fewer than 1000 indigenous malaria cases increased from 13 in 2000 to 33 in 2015 [7], and six countries (United Arab Emirates, Morocco, Armenia, Turkmenistan, Kyrgyzstan and Sri Lanka) achieved at least 3 consecutive years of zero indigenous cases between 2000 and 2015 and were certified as malaria free by WHO [8].

*Plasmodium* species infecting humans share a similar life cycle with an initial development phase in the liver and subsequent further proliferation in the blood of the host. They also show a similar susceptibility to some antimalarial drugs such as quinine, chloroquine and artemisinin, as well as the development of resistance to these drugs [9, 10]. Transmission is also mediated by the same group of anopheline mosquitoes [11]. *P. vivax* malaria can relapse after chemotherapy with drugs that kill the parasites only in the intraerythrocytic development stage [12], but 8-aminoquinolines such as primaquine are known to prevent this effectively [13]. Thus, systematic control programmes involving appropriate chemotherapy including administration of primaquine to patients with *P. vivax* malaria, as well as proper mosquito control, can simultaneously reduce the number of malaria incidents caused by any *Plasmodium* species.

There have been reports of malaria in humans caused by other *Plasmodium* species that naturally infect other primate hosts. But zoonotic malaria is rare, except for that caused by *P. knowlesi*, which naturally infects macaque monkeys such as the long-tailed and the pig-tailed macaques (*Macaca fascicularis* and *M. nemestrina*, respectively). Human infection by *P. knowlesi* has been reported since the 1960s [14, 15], but it had been long thought exceptional like other zoonotic malarias.

The NMCP rolled out in Malaysia in the 1960s had achieved a dramatic reduction of the number of human malaria cases caused by *P. falciparum* and *P. vivax* in Sarawak in Malaysian Borneo by the late 1990s–early 2000s. By contrast, the incidence of *P. malariae* which is rather rare in that state [16] showed an apparent increase [17]. At that time, the species of *Plasmodium* present in patients in Sarawak was identified solely by microscopical observation of parasites in blood films. However, a molecular diagnosis combining nested PCR and DNA sequencing revealed that most of the malaria cases attributed to *P. malariae* by microscopy were actually caused by *P. knowlesi* [18]. Following the identification of human cases of *P. knowlesi* in Sarawak, similar cases were identified in neighbouring Sabah state [19] and within the Peninsular Malaysia [20]. Human infections of *P. knowlesi* have also been reported in other South East Asian countries including Vietnam and Thailand [17], and *P. knowlesi* malaria is now recognised as the fifth human malaria [21]. It has been confirmed that *P. knowlesi* can produce gametocytes in patients who naturally acquire the infection [22], although transmission of *P. knowlesi* malaria between humans has not been reported yet. The clinical importance of *P. knowlesi* malaria is particularly high in Malaysia, where malaria caused by the other four human-infective species has been almost completely eliminated thanks to the successful NMCP [6]. Now, almost all malaria cases identified in the country are caused by *P. knowlesi*.

Like *P. knowlesi*, other *Plasmodium* species that naturally infect non-human primates have been considered as potential threats to human health through zoonosis [23, 24]. Macaques in Sarawak in Malaysian Borneo are known to be the reservoir of six *Plasmodium* species—*P. knowlesi*, *P. inui*, *P. cynomolgi*, *P. coatneyi*, *P. fieldi* and *P. simiovale* [25]. Of these, *P. cynomolgi* has been proven to naturally cause human infection [26], and *P. inui* can establish an infection when experimentally introduced into humans [27]. Clinical cases of zoonotic malaria caused by these species are currently either extremely rare (*P. cynomolgi*) or unreported (*P. inui*), but they might become the next *Plasmodium* species to significantly affect human health in the future.

Zoonotic malaria has been reported in South America as well. The species of *Plasmodium* implicated there are *P. simium* [28] and *P. brasilianum* [29], parasites that naturally infect platyrrhine monkeys. The two species, *P. simium* and *P. brasilianum*, have been shown to be phylogenetically very close to *P. vivax* and *P. malariae*, respectively [30].

## Malaria and *Plasmodium* biology

### Life cycle of *Plasmodium*

All *Plasmodium* species share a similar life cycle [31]. It has two parts—in the first, the parasite infects a person (or a vertebrate host), and in the second, it is transmitted from the malaria patient (or infected vertebrate host) to another host by an insect vector. The vectors that transmit the five *Plasmodium* species naturally infecting humans are mosquitoes of the genus *Anopheles*. These mosquitoes also transmit other *Plasmodium* species parasitising other mammals whereas transmission of *Plasmodium* infecting birds and reptiles depends on mosquitoes of other genera or other blood-sucking insects [32].

The *Plasmodium* life cycle begins when parasites known as sporozoites produced in the insect vector enter the blood of the vertebrate host following a bite [33]. Sporozoites deposited in the dermis [34] rapidly migrate to the liver and invade hepatocytes where they multiply by thousands—a process known as schizogony [35]. The resulting parasites, now called merozoites, are released back into the blood [36] and infect erythrocytes. In an erythrocyte, one merozoite multiplies asexually by schizogony to generate between 8 and 64 new merozoites (the number depending on the species) [37]. These new merozoites are released back to the blood, and the parasites repeat this intraerythrocytic propagation cycle every 24 (*P. knowlesi*), 48 (*P. falciparum*, *P. ovale*, *P. vivax*) or 72 (*P. malariae*) hours. Some merozoites then differentiate into the next developmental stage called the gametocyte for sexual reproduction [38, 39]. Just when gametocyte differentiation (gametocytogenesis) starts depends on the species. For example, *P. falciparum* needs to complete several cycles of intraerythrocytic propagation before it starts differentiation into gametocytes, whereas *P. vivax* continuously produces gametocytes even in its early intraerythrocytic propagation cycles (Table 1). While each gametocyte has a similar appearance during its early development, they are already programmed to differentiate into either male or female gametes (gametogenesis, sexually committed). This completes the part of the parasites’ life cycle that occurs inside the human body. Development beyond the gametocyte stage normally takes place following a blood meal, in the lumen of the mosquito midgut, where the male and the female gametes fuse [52]. However, there are sporadic reports of exflagellated forms (male gametes) of *P. falciparum* observed in the human body [53].

**Table 1:** Onset of gametocyte production and recurrence in human malaria

Causative species	Onset of gametocyte production	Recurrence	
		Relapse	Recrudescence
*P. falciparum*	After several rounds of intraerythrocytic asexual reproduction (“gametocytes may make their appearance in small numbers on or about the 10th day following the first day of fever, and their numbers increase rapidly day by day for 2 or 3 weeks” [40])	Unknown (hypnozoites not observed yet)	Known to occur (latent period usually < 2 months, but can be > 2 years [41])
*P. vivax*	Continuous from early rounds of intraerythrocytic asexual reproduction (“sexual forms may occur as early as the 6th or 7th day, reaching their maximum number on or about the 10th day” [40])	Well documented (latency period ranges from < 2 weeks to > 1 year and varies systematically by geographic region [42]; hypnozoites in liver observed [43]; estimated to be majority of recurrence [44])	Unknown
*P. malariae*	Probably continuous from early rounds of intraerythrocytic asexual reproduction (after a prepatent period (16–59 days [45]), gametocytes emerges in patient’s blood together with intraerythrocytic parasites [46])	Unknown (hypnozoites not observed yet)	Known to occur (latent period can be > 40 years [47])
*P. ovale*	Continuous from early rounds of intraerythrocytic asexual reproduction (“[gametocytes] appear in the peripheral blood a little earlier than in B.T. [benign tertian]” [40])	Clinical cases reported [48]; molecular evidence for a causal relationship between dormant liver stages and subsequent relapses unavailable [49]; hypnozoites not observed yet [50]	Unknown
*P. knowlesi*	Unknown (gametocytes identified in some of the naturally infected malaria patients [22])	Unknown (hypnozoites not observe [51])	Unknown

The second part of the life cycle in the insect vector begins when the insect ingests the blood containing gametocytes from an infected vertebrate host. The gametocytes are activated once exposed to the specific environment of the mosquito midgut lumen, and the male and the female gametocytes differentiate to produce microgametes and macrogametes, respectively [52]. The microgamete fertilises the macrogamete to produce a zygote, the only developmental stage of the parasite that has a diploid genome [54]. Genetic crossing experiments with gametocytes of two clones of *P. falciparum* with different allelic variants demonstrated that recombination can occur in zygotes [55, 56]. Soon the zygote undergoes meiosis and differentiates into a motile form, the ookinete, that now contains four haploid genomes in its nucleus [54]. The ookinete penetrates the wall of the mosquito midgut and forms an oocyst on the outer side [57]. In the oocyst, several rounds of mitosis take place, and numerous sporozoites are produced by sporogony [58, 59]. When the oocyst matures, it ruptures, and sporozoites released into the haemolymph migrate to the salivary glands, where they acquire the ability to infect human cells [60] when released into the body of a vertebrate host during a blood meal. Human-infecting *Plasmodium* species complete this second part of the life cycle (gametocytes to sporozoites ready to infect the next person) in around 10–18 days.

Besides the nucleus, the *Plasmodium* cell has two distinct organelles that contain their own genomic DNA, the mitochondrion and the apicoplast (see below). It has been shown that each oocyst of *P. falciparum* developing in the mosquito inherits the mitochondrial genomic DNA uniparentally from the female gamete [61]. A further study reported that both organellar genomic DNAs were detected in female gametes of *P. gallinaceum* (a species infecting chickens) but not in male gametes [62]. This suggests that *Plasmodium* inherits both the mitochondrion and the apicoplast only through the female gamete (macrogamete).

### Recurrence of malaria and the hypnozoite

Malaria can recur after the parasites apparently have been cleared from the patient’s blood. Recurrence is due either to a recrudescence or a relapse (Table 1). Recrudescence originates from a minor population of parasites that survived undetected in the patient’s blood, whereas relapse is caused by cryptic, dormant cells called hypnozoites [43] that persist in the patient’s liver. Hypnozoites originate exclusively by differentiation from sporozoites and never from another form of the parasite, such as the merozoites circulating in the patient’s blood [63].

*P. vivax* is known to produce hypnozoites that cause relapses after the parasites have been cleared from the patient’s blood by treatment with antimalarial drugs such as chloroquine or quinine [12]. There is one report that, in the majority of relapse cases studied, the genotype of the parasites in the first relapse is different from those during the preceding acute episode [64]. This result was probably due to the following: (1) *P. vivax* sporozoites of two or more different genotypes infecting a person, (2) sporozoites other than the one that caused the acute episode differentiating into a hypnozoite in the liver, and (3) only one of those hypnozoites causing the relapse event. External stimuli such as other infections, including *P. falciparum* malaria, have been suggested to activate the hypnozoite and initiate a relapse of *P. vivax* malaria [65], although the mechanism has not been explained.

Of the other human malaria parasites, *P. ovale* has long been believed to develop hypnozoites because there are reports of recurrence without a second infection by the same species. However, this view has been questioned recently because of a lack of experimental and clinical data unequivocally supporting the presence of hypnozoites in the liver [49, 50].

Neither *P. falciparum* nor *P. malariae* is thought to develop hypnozoites, and such cells have never been identified in either species [63]. Nevertheless, these two species can cause persistent infection without the development of any symptoms over long periods of time. For example, there is a case report of a *P. falciparum* infection that persisted asymptomatically in the human body for 13 years [66]. A *P. malariae* case caused by blood transfusion from a donor who had probably acquired the infection over 40 years ago has also been reported [47]. *Plasmodium* parasites can be maintained in the human blood over long periods of time at a very low number when their growth rate and the host’s immunity are able to maintain a subtle balance. Recurrence (recrudescence) is believed to begin when the balance is broken.

Whilst there is strong evidence that hypnozoites cause relapses in *P. vivax* malaria, some recurrences of *P. vivax* malaria might have originated from non-hypnozoite cells, as in other human malarias [67–69]. Recently, it was reported that recurrence of parasitaemia had been recorded in some neurosyphilis patients who had received a *P. vivax* malaria patient’s blood for malariotherapy [70–72]. Because *P. vivax* does not persist as sporozoites in human blood, no hypnozoite could have developed in the recipients. Thus, these records may suggest that *P. vivax* malaria can recur independent of hypnozoites.

### Gametocytes

Throughout development in their vertebrate hosts, *Plasmodium* cells have a haploid genome. Nevertheless, a cloned line of *Plasmodium* originating from a single cell generates both male and female gametocytes in the vertebrate host. This indicates that the sex of *Plasmodium* gametocytes is not determined chromosomally but epigenetically, and evidence explaining the mechanism is being accumulated [38]. The gametocyte sex ratio is apparently affected by environmental factors [73–75], and this may optimise parasite transmission.

Gametocytes of the *Plasmodium* species that infect humans are known to be susceptible to 8-aminoquinolines such as primaquine, but not to other antimalarials such as artemisinin and chloroquine that kill the parasites in asexual intraerythrocytic development [76]. Thus, even after parasites in their asexual intraerythrocytic development cycle are removed by treatment with those antimalarials, transfer of gametocytes in the blood can cause malaria in other people. In addition, it has been reported that *P. falciparum* gametocytes can persist for several weeks after the clearance of asexual blood stage parasites by drug treatment such as artemisinin-combination therapy [77]. An in vitro study using culture of gametocytes from one clinical isolate and two laboratory strains of *P. falciparum* observed that gametocytes had a 50% survival rate of 2.6–6.5 days and that surviving cells were detectable until almost 2 months after the start of the experiment [78]. Both the rate of clearance of gametocytes from the patient’s body and the rate of decline of gametocyte infectivity for mosquitoes depend on multiple factors such as the kind of treatment and the way it is implemented [79, 80], as well as host immunity [81]. People with asymptomatic infection can carry a high number of gametocytes in their blood, just like patients with symptoms of malaria [82–84]. Therefore, gametocytocidal treatment should be given not only to malaria patients with symptoms but also to asymptomatic *Plasmodium* carriers to prevent the parasites in them causing new malaria cases.

### Asymptomatic carriers

In areas endemic for malaria, many people carry *Plasmodium* without developing symptoms because of acquired immunity [85, 86]. Often, the presence of parasites in those carriers cannot be detected either by microscopy or rapid detection tests (RDTs), the two standard tests for detecting *Plasmodium* in malaria patients’ blood. However, infection can be detected with more sensitive methods, e.g., molecular detection by PCR and LAMP [87], or ultrasensitive variations of RDTs [88]. Asymptomatic carriers can supply infectious gametocytes to mosquitoes, though their impact on malaria in the community where they live may be low or negligible when the number of gametocytes in the blood is not high [89]. Asymptomatic *Plasmodium* carriers can develop an episode of malaria when their immunity against the parasite is compromised, for example, when they move to a malaria-free area where they no longer have new *Plasmodium* infections that sustain immunity against the parasite. As a result, asymptomatic carriers may cause imported malaria [90, 91]. Even without developing any episodes of malaria, they can also cause transfusion malaria [92] or organ-transplantation malaria [93].

The importance of controlling asymptomatic carriers of malaria parasites in the modern world is higher than ever. It is mainly because people’s movements have become much easier than in earlier times, thanks to economic development and transportation. In addition, the number of refugees from regional conflicts, many of which occur in malaria endemic areas, has increased. Asymptomatic carriers within migrants from malaria endemic areas ought to be identified and adequately cared for, like patients with apparent malaria symptoms, in order to prevent the spread or re-introduction of malaria into malaria-free areas [94, 95].

### Apicoplast and plant-like metabolism

The genus *Plasmodium* belongs to a larger group of protozoans called the Apicomplexa, part of the superphylum Alveolata that also includes dinoflagellates and ciliates [96]. Like *Plasmodium*, almost all apicomplexans are obligate parasites [31], and many of them, including all *Plasmodium* species, have a vestigial, non-photosynthetic plastid called the apicoplast in the cell [97–99]. The apicoplast is a secondary plastid surrounded by four layers of membrane [100, 101] and has a tiny genome, the smallest in size of all known plastid genomes [102, 103]. Recently, exceptional apicomplexan species that have a photosynthetic plastid were also discovered [104, 105]. These new species, grouped as chromerids, can grow phototrophically without parasitising other organisms. The plastids of chromerids contain chlorophyll *a* but lack chlorophyll c that is universally found in the plastids of other phototrophic alveolates. Like the apicoplast, the photosynthetic plastids of chromerids are secondary plastids [106]. Features of the organellar genome suggest that all apicomplexan plastids originated from the last common ancestor of present apicomplexans [107].

Unlike plastids of photosynthetic organisms, the apicoplast is non-photosynthetic, and almost all gene products encoded in the tiny organellar genome are predicted to be involved in either transcription or translation [108]. Therefore, the reason why parasitic apicomplexans such as *Plasmodium* carry a non-photosynthetic plastid was not clear when the organelle’s presence in the parasite was first recognised [109]. However, as the genomic information encoded in the nuclear genome of the parasites became available [110], it gradually became evident that the *Plasmodium* apicoplast is involved in plant-type metabolic pathways including isoprenoid biosynthesis [111], type II fatty acid biosynthesis [112] and haem biosynthesis [113]. These plant-type pathways involving the apicoplast have been suggested to be important for *Plasmodium* to complete its development, especially in mosquitoes [114, 115] and in the liver [116, 117]. However, it has been shown that *Plasmodium* in the intraerythrocytic cycle can keep growing in in vitro culture as long as a sufficient amount of isopentenyl pyrophosphate (IPP) is supplied in the culture medium, even when they have lost the apicoplast [118]. This suggests that the requirement of the apicoplast for this stage of the parasites is solely to obtain IPP. IPP is the precursor of various isoprenoids and is essential for eukaryotes including *Plasmodium* to survive, and the plant-type methylerythritol phosphate (MEP) pathway in the apicoplast is the only source of this critical molecule in the parasite [119]. One of the enzymes involved in the MEP pathway, 1-deoxy-D-xylulose-5-phosphate reductoisomerase, is inhibited by a specific inhibitor called fosmidomycin [120], and growth of *P. falciparum* is inhibited by fosmidomycin both in vitro and in vivo [121, 122]. By contrast, fosmidomycin does not show a significant inhibitory effect on the growth of another distantly related apicomplexan *Toxoplasma gondii*, which also is supposed to depend on IPP supplied by the MEP pathway in the apicoplast [123]. The molecular basis of the resistance of *T. gondii* to fosmidomycin has been studied, and it was suggested that poor drug uptake through the parasite’s plasma membrane is the cause [124]. Another study suggested that the uptake of fosmidomycin into the erythrocyte which *P. falciparum* infects depends on a new permeability pathway induced by the parasite [125]. Although fosmidomycin has good properties as a novel antimalarial [126], *Plasmodium* may be able to develop resistance to the drug by mutation in unexpected genes.

Some apicomplexans such as *Cryptosporidium* and *Gregarina* do not have the apicoplast [127], while *P. falciparum* and *T. gondii* cannot survive if they lose the organelle [118, 128]. The enzymes involved in the plant-like metabolism in the apicoplast are encoded in the nuclear genome, and apicomplexan species that do not have the apicoplast tend to lack all the genes specifying these enzymes [129]. By contrast, the genome of *Cryptosporidium* species encodes a remarkably large number of putative amino acid transporters compared to apicomplexans with an apicoplast [130]. These species without an apicoplast probably acquire the metabolites that ordinary apicomplexans synthesise in the apicoplast, from the host using some of those additional transporters.

### Antimalarial drugs and resistance

From ancient times, various plant products have been used in folk medicine to treat malaria. In the seventeenth century, it was shown that the bark of South American quina-quina trees (*Cinchona* spp.) contains an agent with antimalarial activity. This substance, quinine, has been used in the treatment of malaria since then [131]. In the nineteenth century, efforts began to chemically synthesise pure compounds with antimalarial activity, and some clinically useful synthetic antimalarials such as chloroquine became available in the 1930s. In 1972, another natural compound used traditionally in China, artemisinin, was reported to have antimalarial effect.

Antimalarial drugs currently used in the clinical treatment of human malaria come in five classes based on their structural backbone and apparent action [132]. Those classes are (1) endoperoxides (e.g. artemisinin and its derivatives), (2) 4-aminoquinolines (chloroquine), aryl-amino alcohols (quinine, mefloquine), (3) antifolates (pyrimethamine, proguanil, sulfadoxine), (4) naphthoquinones (atovaquone) and (5) 8-aminoquinolines (primaquine, tafenoquine). The main targets of inhibition by 4-aminoquinolines, anitifolates and naphthoquinones have been shown to be detoxification of haem released from digested haemoglobin, pyrimidine biosynthesis and mitochondrial cytochrome *b* involved in oxidoreduction, respectively. Aryl amino alcohol class inhibitors seem to inhibit the same metabolism as 4-aminoquinolines, whereas endoperoxides, such as artemisinin, act on multiple cellular processes involving reactive oxygen species in *Plasmodium* cells.

The parasites causing human malaria, especially *P. falciparum*, have acquired resistance to each of these antimalarials one by one, and today, drug-resistant parasites are prevalent in malaria endemic areas [133]. It has been well documented that point mutations causing amino acid substitutions in the active site of dihydrofolate reductase (the target enzyme of pyrimethamine and proguanil) make *P. falciparum* highly resistant to those antifolate drugs [134, 135]. Another example includes specific point mutations occurring in *pfcrt* and *pfmdr1*, which specify transporters CRT and MDR1, respectively, which promote the efflux of antimalarials such as 4-aminoquinolines and aryl amino alcohols from the digestive vacuoles of *P. falciparum* [136]. As a result, parasites with these mutated genes become resistant to antimalarials that block haem detoxification in the digestive vacuoles. Point mutations in a gene are not the only the way for the parasites to acquire resistance to antimalarials. For example, when expression of the affected gene product is elevated because of gene amplification or a change in regulatory mechanisms, antimalarials can reduce or lose their efficacy [137, 138]. It is also potentially possible that *Plasmodium* species acquire new machinery with which the parasites can survive without the metabolism targeted by an antimalarial. For example, if *Plasmodium* acquires a new transporter with which they can acquire IPP from the host, as *Cryptosporidium* species can, the parasites become resistant to fosmidomycin.

It has been reported that clinical isolates of *P. falciparum* showing resistance to multiple antimalarial drugs tend to have defects in DNA mismatch repair [139, 140]. Genetic changes are often harmful, so having defects in DNA mismatch repair, which causes a higher rate of genetic changes in the genome, can reduce the fitness of the parasites affected. However, parasites with such defects have a potential to generate a wider variety of genetic changes compared to those without such defects. This is presumably beneficial for the parasites in order to survive under strong drug pressure. The A + T content of the genome of *Plasmodium* species is generally high (> 60%); it is especially high in the genome of *P. falciparum*—nearly 80% in coding regions and approaching 90% in non-coding regions. The genome of *P. falciparum* has been shown to be prone to small genetic changes such as indel mutations [141]. The high A + T content of the genome may also contribute to *Plasmodium* developing drug resistance.

### Host specificity

The natural host range of *Plasmodium* depends on the species. It can be extremely narrow as in *P. falciparum*, which infects humans but not African apes that are phylogenetically very close to humans [142]. The range can also be very wide as in *P. relictum*, which is known to infect more than 100 different species of birds around the world, classified into different families and orders [143]. Traditionally, *Plasmodium* species are sub-categorised into distinct subgenera based on their morphology and ranges of vertebrate hosts and vectors [32]. It has been pointed out that the genus *Plasmodium* as a whole is polyphyletic and that the taxonomy of the order Haemosporidia, which consists of *Plasmodium* and other related genera, has many conflicts [144]. Nevertheless, each subgenus of *Plasmodium* seems to be monophyletic. *Plasmodium* species that infect mammals generally belong to either one of three subgenera, *Laverania*, *Plasmodium* or *Vinckeia*, apart from some species recently identified from ungulate hosts [32, 144, 145]. The subgenera *Laverania*, *Plasmodium* and *Vinckeia* consist of parasite species that infect apes, monkeys and rodents, respectively.

Unlike *Plasmodium* species that infect birds, which are transmitted by a wide variety of mosquitoes including *Culex* and *Aedes*, mammalian malaria parasites belonging to subgenera *Laverania*, *Plasmodium* and *Vinckeia* are transmitted only by anopheline mosquitoes [32]. This is because mammalian *Plasmodium* species can complete their development from gametocytes to infectious sporozoites only in anopheline mosquitoes. However, this does not mean that these parasites cannot developmentally differentiate from gametocytes in non-anopheline mosquitoes [146]. There was an early report that oocysts formed on the midgut epithelium, and sporozoites were observed in the salivary gland when *Culex bitaeniorhynchus* was fed with human blood containing gametocytes of *P. falciparum* or other human malaria parasites [147]. However, another recent study reported that a laboratory strain of *P. falciparum* fed to *C. quinquefasciatus* developed ookinetes that soon lysed and never formed oocysts [148]. This suggests that *P. falciparum* is killed by the mosquito’s immune system when the ookinetes are exposed to the haemolymph of non-anopheline mosquitoes [149].

It is not true that all anopheline mosquitoes are equally important in transmitting *Plasmodium* between humans or animals; different *Anopheles* species have different habitats, feeding behaviour and preference for the animal species from which they suck blood [150]. These differences may promote species differentiation in the parasites they carry and provide a chance for the parasite to switch its host [151].

Of the four human *Plasmodium* species, *P. falciparum* belongs to subgenus *Laverania*, whereas all the others belong to subgenus *Plasmodium*. *P. knowlesi* that causes zoonotic malaria in humans also belongs to the subgenus *Plasmodium*. Generally, *P. falciparum* only infects humans in natural conditions [152], though it is possible to adapt the species to infect chimpanzees in the laboratory [153]. Krief et al. reported that they isolated *P. falciparum* from bonobos (*Pan paniscus*) kept at the Lola ya Bonobo Sanctuary in the Democratic Republic of the Congo, though the mitochondrial haplotype map they drew indicated that the isolates from bonobos were genetically distant from *P. falciparum* parasites infecting humans [154]. The non-*P. falciparum Laverania* species that is phylogenetically closest to *P. falciparum* is *P. praefalciparum*, which infects gorillas but not humans or chimpanzees [152]. As with these two species, all *Plasmodium* species of subgenus *Laverania* so far described show a strong host specificity in their natural transmission [152, 155]. A longitudinal survey of anopheline mosquitoes carried out in two wildlife reserves in Gabon where different *Laverania* species coexist revealed that the three sylvan *Anopheles* species collected in the survey, *An. vinckei*, *An. moucheti* and *An. marshallii*, carried multiple *Laverania* species whose vertebrate host specificity varied [151]. This indicates that the strong host specificity of the *Laverania* species is not solely caused by specific association between anopheline vectors and vertebrate hosts. A recent comparative genomics study between *Laverania* species revealed that these parasites have striking copy number differences and structural variations in multiple gene families in their genomes [156]. Variations in the *stevor* family, which has been shown to be involved in host-parasite interactions in *P. falciparum* [157], showed a host-specific sequence pattern. Probably these variations are critically important for each *Laverania* species to determine their strong host specificity. *Rh5*, a member of another gene family, has been shown to be important for *Plasmodium* species of subgenus *Laverania* to bind to erythrocytes of specific hosts [158]. *EBA165*, a member of the erythrocyte binding-like (EBL) gene family, is pseudogenised in the genome of *P. falciparum* but not in other *Laverania* species’ genomes. A recent study showed that *P. falciparum* becomes capable of binding to ape erythrocytes but loses the ability to bind human erythrocytes when a functional *EBA165* product is expressed [159]. This suggests that losing the functional *EBA165* product was a key step in the emergence of the human-infecting *P. falciparum* from its *P. praefalciparum*-like ancestor that probably did not infect humans*.*

In the mosquito survey in Gabon described above, it was also found that the three sylvan species of Anopheles carried *Plasmodium* species which were extremely close to *P. vivax* or *P. malariae* [151]. In a phylogenetic analysis, these *P. vivax*-like isolates from mosquitoes collected in the survey in Gabon made a cluster with *P. simium*, one of the two South American zoonotic *Plasmodium* species [151]. Another study suggested that *P. brasilianum*, the other South American zoonotic *Plasmodium*, is probably an amphixenotic variant of *P. malariae* that acquired infectivity to the simian hosts while also keeping its original human infectivity [29]. All these suggest that both *P. vivax* and *P. malariae* can switch their host more easily than the species of subgenus *Laverania* [151]. However, the mechanisms behind host switching of species of subgenus *Plasmodium* including *P. knowlesi* and other simian species causing zoonotic malaria are not well understood.

## Conclusions

Humans have long suffered from malaria, the disease caused by *Plasmodium*. Thanks to the discovery of natural and chemically synthesised antimalarials, malaria has become a disease curable by chemotherapy. Together with mosquito vector control using insecticides, treatment of malaria patients with synthetic antimalarials such as chloroquine and artemisinin has dramatically reduced the burden of malaria in the modern world compared to the past. Nevertheless, malaria is still prevalent, killing hundreds of thousands of people globally every year.

One of the problems that hinder control of malaria is the emergence and spread of chemotherapy-resistant parasites [160–162]. To solve this problem, novel antimalarial substances whose targets are different from those of existing antimalarials are sought. Using those inhibitors in combination with existing antimalarials largely reduces the risk that the parasites acquire resistance to each substance. For example, inhibitors of the plant-like metabolic pathways of the parasite are attracting attention.

Another problem is that *Plasmodium* can be easily carried from endemic areas to non-endemic areas in the modern world because of increased movement of people. Malaria-free countries and areas are steadily increasing, but imported malaria is a commonly shared threat against health in many countries and areas. *Plasmodium* species can be retained in the human body for a long time without causing any overt symptoms. Asymptomatic *Plasmodium* carriers can start developing malaria spontaneously and spread imported malaria in malaria non-endemic countries and areas. These people may also spread the parasite when they are involved in blood transfusions and organ transplantation as donors.

*P. falciparum* and other human-infecting *Plasmodium* species share a characteristic that lets them infect humans, which is the outcome of convergent evolution. Currently, *Plasmodium* species that naturally cause malaria in humans are limited, but other species may also acquire natural infectivity to humans and start causing new zoonotic malaria at any time. The physical distance between humans and non-human animals such as other primates can depend on the degree of local development, and this could affect the chance of non-human *Plasmodium* species becoming the cause of zoonotic malaria in humans.

*Plasmodium* species may change their insect hosts as well. Unlike the *Plasmodium* species that infect mammals, avian malaria parasites develop and produce infectious sporozoites in non-anopheline mosquitoes. This implies that mammalian *Plasmodium* species also could acquire resistance to the immune system of non-anopheline mosquitoes such as *Culex* and *Aedes* and use them as transmission vectors. Some non-anopheline mosquito species can breed even in harsh environments such as in tunnels, on the coast or in urbanised areas, and can be cosmopolitan [163, 164]. If a *Plasmodium* species that can cause malaria in humans acquired the capability of completing its mosquito stage development in non-anopheline mosquitoes, its impact on human society could be substantial.

The relationship between humans and *Plasmodium* changes dynamically due to both the parasites’ nature and the activities of humans. Understanding the basic biology of the parasites that leads to these changes, and applying the knowledge to malaria control, should help to achieve a healthier global society.

## Data Availability

Not applicable.

## Abbreviations

ITN: Insecticide-treated bed net
NMCP: National Malaria Control Programme
RDT: Rapid detection test
IPP: Isopentenyl pyrophosphate
MEP: Methylerythritol phosphate
