# Correction to: *Plasmodium*—a brief introduction to the parasites causing human malaria and their basic biology

**DOI:** 10.1186/s40101-021-00254-0

**Published:** 2021-01-29

**Authors:** Shigeharu Sato

**Affiliations:** 1 Borneo Medical and Health Research Centre, Faculty of Medicine and Health Sciences, Universiti Malaysia Sabah, Jalan UMS, 88400 Kota Kinabalu, Sabah Malaysia; 2 Department of Pathobiology and Medical Diagnostics, Faculty of Medicine and Health Sciences, Universiti Malaysia Sabah, Jalan UMS, 88400 Kota Kinabalu, Sabah Malaysia

**Correction to: J Physiol Anthropol 40, 1 (2021)**

**https://doi.org/10.1186/s40101-020-00251-9**

It was highlighted that the original article [1] contained an incorrect sentence in the fourth paragraph of the section Recurrence of malaria and the hypnozoite. This Correction article shows the incorrect and correct sentence. The original article has been updated.

**Incorrect sentence**

A *P. malariae* case caused by blood transfusion from a donor who had probably acquired the infection over 40 years ago has also been reported.

**Correct sentence**

A *P. malariae* case that most likely developed after an asymptomatic infection lasted over 40 years has also been reported.
